# Magnitude of Preterm Birth and Its Associated Factors: A Cross-Sectional Study at Butajira Hospital, Southern Nations, Nationalities, and People's Region, Ethiopia

**DOI:** 10.1155/2020/6303062

**Published:** 2020-06-11

**Authors:** Ritbano Ahmed Abdo, Hassen Mosa Halil, Muhammed Abdu Muhammed, Mohammed Sultan Karebo

**Affiliations:** ^1^Department of Midwifery, College of Medicine and Health Sciences, Wachemo University, Hossana, Ethiopia; ^2^Department of Midwifery, College of Health Sciences, Samara University, Samara, Ethiopia; ^3^Department of Statistics, College of Natural and Computational Science, Wachemo University, Hosanna, Ethiopia

## Abstract

**Background:**

Preterm birth infants are at a greater risk of mortality and a variety of health and developmental problems; reliable data support that this rate is increasing in almost all countries. The purpose is to find the magnitude of preterm birth and its associated factors among newborns delivered at Butajira Hospital, Southern Nations, Nationalities, and People's Region, Ethiopia.

**Methods:**

This hospital-based cross-sectional study was carried out on 304 maternity cards using the systematic sampling method during May 1^_^21 in 2019. The data collection was performed using a structured case record form. The data were entered into EpiData software (version 3.1) and analyzed using SPSS software (version 21). Binary and multivariable logistic regression analyses were computed to identify the associated factors at 95% CI.

**Results:**

Overall magnitude of preterm birth was observed to be 15.5%. The factors significantly associated with the preterm birth were preeclampsia (AOR = 4.0; 95% CI: 1.6-10.0), and premature rupture of fetal membranes (AOR = 4.2; 95% CI: 1.4-12.9).

**Conclusion:**

Preterm birth is still public concern in the study area. The concerned administrative body should implement health education programs and improve the quality of health care delivered to pregnant mothers to control these associated factors and, consequently, promote public health in the study area.

## 1. Introduction

The World Health Organization (WHO) defines a preterm birth as birth that occurs before 37 completed weeks of gestation, or fewer than 259 days from the first date of a woman's last menstrual period [1]. Preterm infants are at a greater risk of mortality and of having a variety of health and developmental problems [2]. Fifteen million babies are born prematurely worldwide each year [3] and more than 80% of these births occur in Asia and Sub-Saharan Africa. Reliable data reveals that this rate is increasing in almost all countries [4, 5]. However, the rate can vary widely in different countries. For example, preterm births make up 13.4% of deliveries in North Africa compared to 8.7% in Europe [5].

Prematurity has become the leading cause of newborn mortality worldwide, resulting in more than one million deaths each year. Responsible for 35% of worldwide neonatal deaths, the condition is the second leading cause of death among children under five after pneumonia [3, 5]. In Ethiopia, 320,000 babies are born early each year and 24,400 children under five die directly due to preterm complications [6]. Additionally, institution-based studies conducted in Ethiopia show a high rate of neonatal death due to preterm birth [7, 8]. Those newborns who initially survive will face both short and long-term complications. Short-term complications include acute respiratory, gastrointestinal, immunologic, and central nervous system problems. Long-term complications of preterm birth include motor, cognitive, visual, hearing, behavioural, social-emotional, health, and growth problems. These issues may not become apparent until later years though may become clear and persist well into adulthood [9]. Due to this, a preterm birth could have a significant and negative financial consequence on the family of the infant [10, 11].

A variety of factors can contribute to preterm birth, including history of previous preterm birth [12, 13], pregnancy induced hypertension [12, 14, 15] premature rupture of foetal membranes, [14, 16, 17] multiple pregnancy, bleeding during pregnancy [12, 18], history of abortion [14, 18, 19], foetal malformation [14, 15, 20], inadequate antenatal care (less than four visits) follow up [13, 15, 16], polyhydramnios [17, 18], previous caesarean section [14], and rural residence [19].

At present, local data is crucial to implement an intervention in order to reduce the risk of preterm birth. In Ethiopia, as observed in different studies from across the country, the prevalence of preterm birth is estimated to be between as low as 4.4% [19] and as high as 25.9% [16]. The geographical distribution and other factors associated with preterm birth vary across different cultures and socioeconomic statuses within a society. Therefore, the results gained from a study of one area might not be relevant to other areas [14, 19, 21, 22]. With this in mind, this study has endeavoured to find the magnitude of preterm birth and its associated factors of preterm birth among newborns delivered at Butajira Hospital, Southern Nations, Nationalities, and People's Region, Ethiopia.

## 2. Methods and Materials

### 2.1. Study Design, Setting, Period, and Population

A hospital-based cross-sectional study was carried out in Butajira Hospital from May 1 to 21, 2019. The study populations for the study were randomly chosen maternity cards for mothers who had given birth at the Butajira Hospital in the previous year prior to the data collection period.

### 2.2. Sampling Size and Sampling Procedure

The sample size of 304 was calculated using a single population proportion formula with the following assumptions; the proportion of preterm birth was taken from the study conducted in Jimma University Specialized Teaching and Referral Hospital of 25.9% [16], with a 95% confidence interval, margin of error of 5%, and 10% missed-items. A systematic random sampling technique was used to select the study subjects (cards).

### 2.3. Data Collection Tool, Procedure and Quality Control

A pretested case record form was used to review data from maternal cards, which was adapted from other related researches [13–23]. Midwives, two with diplomas and one with a bachelor's degree, were hired for the data collection and supervision. The quality of data was maintained through; a data collection form was pretested on 5% of a calculated sample size before the actual data collection period in the Workable Comprehensive Hospital. The data collectors were given a one-day intensive instruction on the study tool and data collection. Additionally, the data collectors worked under the close observation of the supervisors to ensure reliability in correct data collection procedures. Moreover, all filled forms were checked daily for completeness, accuracy, clarity, and consistency by the supervisors and the principal investigators. Completeness and consistency of variables during data entry and analysis were checked using frequency distributions.

### 2.4. Data Management and Analysis

The collected data were coded and entered using EpiData version 3.1 software to minimize errors and design skipping patterns. Then, the data were exported and analyzed using SPSS version 24.0. Descriptive statistics, frequency, and proportions were computed to summarise the data. Bivariate analysis between outcome and independent variables was performed separately using binary logistic regression. The strength and direction of the association between an outcome variable and independent variables were expressed in odds ratio through 95% CI. To identify independent factors, the first bivariate logistic regression analyses were carried out to select a candidate for multivariable logistic regression analyses. Multivariable logistic regression analyses were done for variables that have *p* value ≤.25 during the bivariate logistic regression analyses to identify factors associated with the outcome variable and to control for potential confounders. Association between independent and dependent variables were assessed using odds ratio with 95% confidence interval. At the end, *p* < 0.05 was considered as statistically significant in the multivariable model. Hosmer-Lemeshow goodness-of-fit statistic was used to check the necessary assumptions for multivariable logistic regressions was fulfilled.

### 2.5. Operational Definition

Gestational age was estimated based on her last normal menstrual period, using an ultrasound report from chart review.

### 2.6. Ethical Consideration

This study was approved by Wachemo University, research and community services directorate office. Additionally, permission was obtained from the hospital authority before commencing the data collection.

## 3. Result

### 3.1. Socio-Demographic, Obstetric, Medical, and Fetal Characteristics

A total of 304 maternity cards were reviewed in this study, wherein the response rate was 100%. Two hundred forty-two (79.6%) of mothers were aged between 20 and 34 years. The majority of them, 300 (98.7%) were married, while 208 (68.4%) were urban residents. Regarding parity, 193 (63.5%) mothers were multigravida, and the other 28.6% were primipara. Thirty (9.9%) mothers had ever experienced abortion. More than half of the mothers 161 (53%) had four or more antenatal care visits. During their last pregnancy, 28 (9.2%), 10 (3.3%), and 17 (5.6%) of mothers faced the preeclampsia, antepartum haemorrhage, and premature rupture of fetal membranes, respectively.

The majority of the mothers delivered 293 (96.4%) alive neonates. Of these, 27 (9.2%) were low birth weight. According to reports in the mothers' cards, 7 (2.3%) mothers were HIV positive, and in 27 (8.6%) of the mothers, the hemoglobin level was less than 11gm/dl **(**Table 1**)**.

### 3.2. The Magnitude of Preterm Birth

**Figure 1:**
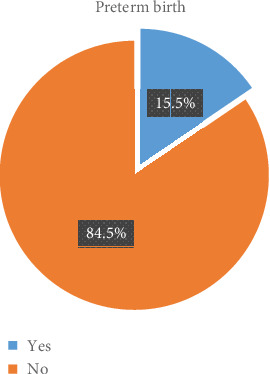
Magnitude of preterm birth in the Butajira Hospital, 2019.

In this study, the overall magnitude of preterm birth was observed to be 47 (15.5%) (Figure 1).

### 3.3. Associated Factors of Preterm Birth

The outcome of bivariate logistic regression analysis showed that mothers aged ≥35 years, being rural residents, with history of previous preterm birth, grand multiparty, and less than 4 antenatal care visits were associated factors for preterm birth. However, in multivariable logistic regression, preeclampsia and premature rupture of fetal membranes were found to be significantly associated with preterm birth. Preterm birth was four times more likely to occur in mothers with preeclampsia in comparison to their counterparts (AOR = 5.1; 95% CI: 2.0, 13.3). Also, mothers with premature rupture of fetal membranes were four or more times more likely to have a preterm birth than their counterparts (AOR = 4.2, 95% CI: 1.4, 12.9) (Table 2).

## 4. Discussion

In this study, the prevalence of preterm birth was observed to be 15.5% and is higher than those of other studies conducted in Brazil, India, Gondar (Ethiopia), and Iran, which were 11.5%, 5.8%, 4.4%, and 5.1%, respectively [17, 20, 21, 23]. This high rate may be due to the fact that the rate of preterm birth is increasing worldwide [3] and that mothers in the abovementioned countries may have better preterm birth prevention rates. In contrast, this study found a lower prevalence of preterm birth compared to other studies in Jimma (Ethiopia) and Kenya, which were 25.9% and 18.3%, respectively [16, 22]. This variation may be due to difference in the study design, study setting, sociocultural status, and implementation of the health-related program. In addition, the prevalence of preterm birth may vary between and within geographical regions [4].

The observed association between preeclampsia during pregnancy with preterm birth is consistent with studies conducted in Iran [12], India [14, 17], Ethiopia [15, 16, 19], and Peru [13]. The reason for this may be mothers with preeclampsia have a greater risk of undergoing preterm birth, as definite intervention of severe preeclampsia terminates pregnancy, thus resulting in preterm.

The finding of this study also revealed that mothers with the premature rupture of fetal membranes were associated with preterm birth. This is comparable with studies done in Iran [12], India [14, 21], and Ethiopia [16, 19]. The reason of these might be due to the fact that labor starts within 24 hours after premature rupture of fetal membranes so increases of preterm labor and preterm birth.

This study understandably shares the limitations of cross-sectional studies. First, as a cross-sectional study, the associations observed between the independent variables and the dependent does not show causal-effect relationship. Secondly, this is a hospital-based cross-sectional study whose findings are not generalized to a general population. Finally, it is important to mention here that data was collected from each mother's card; due to this, some important variables were missing, such as previously highlighted factors with preterm birth in different studies.

## 5. Conclusion

In conclusion, preterm birth is still a public concern in the study area. Preeclampsia and premature rupture of fetal membranes were significantly associated with preterm birth. Therefore, the concerned administrative body should implement health education programs and improve the quality of health care delivered to pregnant mothers to control these risk factors and, consequently, promote public health in the study area.

## Figures and Tables

**Table 1:** Socio-demographic, obstetric, medical, and fetal characteristics in the Butajira Hospital, 2019.

Variables		Frequency (*N* = 304)	Percent
Age group	<20	32	10.5
	20-34	242	79.6
	≥35 and above	30	9.9

Residency	Urban	208	68.4
	Rural	96	31.6

Marital status	Married	300	98.7
	Single	4	1.3

Gravidity	Primipara	87	28.6
	Multipara	193	63.5
	Grand multipara	24	7.9

Ever had abortion	No	274	90.1
	Yes	30	9.9

History of previous preterm birth	No	253	83.2
	Yes	51	16.8

ANC follow-up	Four or above visits	161	53.0
	Less than four visit	143	47.0

Preeclampsia	No	276	90.8
	Yes	28	9.2

Prerupture of fetal membranes	No	287	94.4
	Yes	17	5.6

Antepartum hemorrhage	No	294	96.7
	Yes	10	3.3

Onset of labor	Spontaneous	296	97.4
	Induction	8	2.6

Mode of delivery	Normal vaginal delivery	242	79.6
	Operative vaginal	28	9.2
	Caesarean delivery	34	11.2

Delivery status	Single	280	92.1
	Multiple	24	7.9

Preterm birth	No	257	84.5
	Yes	47	15.5

Sex of fetus	Female	135	44.4
	Male	169	55.6

Fetal status	Alive	293	96.4
	Still birth	11	3.6

Birth weight of live birth baby (*N* = 293)	Normal birth weight (2.5-4 kg)	266	91.8
	Low birth weight (<2.5 kg)	27	9.2

HIV status	Negative	297	97.7
	Positive	7	2.3

Hemoglobin level (prepartum)	≥11g/dl	277	91.1
	<11g/dl	27	8.9

**Table 2:** Associated factors of preterm birth in the Butajira Hospital, 2019.

Variables	Preterm birth		Crude odds ratio (95% CI)	Adjusted odds ratio (95% CI)
	No	Yes		
Residence
Urban	186	22	Reference	Reference
Rural	71	25	3 (1.6, 5.6)∗	1.9 (.8, 4.3)

Age group
<20	29	3	.7 (.2, 2.4)	1.1 (.3, 4.6)
20-34	211	31	1	1
≥35	17	13	5.2 (2.3, 11.8)∗	2.2 (.7, 6.8)

Parity
Primipara	77	10	.8 (.4,1.7)	1.1 (.4, 2.8)
Multipara	166	27	Reference	Reference
Grand multipara	14	10	4.4 (1.1, 10.8)∗	1.5 (.4, 5.1)

Preeclampsia
No	239	37	Reference	Reference
Yes	18	10	3.6 (1.5, 8.4)^∗^	4.0 (1.6, 10.0)^∗∗^

ANC visit in current pregnancy
≥4 visits	143	18	Reference	Reference
<4 visits	114	29	2.0 (1.1, 3.8)∗	1.0 (.4, 2.1)

Premature rupture of fetal membranes
No	248	39	Reference	Reference
Yes	9	8	5.6 (2.1, 15.5)∗	4.2 (1.4, 12.9)^∗∗^

History premature birth
No	222	31	1	1
Yes	35	16	3.3 (1.6, 6.6)∗	2.2 (.9, 5.0)

^∗∗^ *p* < 0.05.

## Data Availability

The datasets used and analyzed during the current study are available from the corresponding author on reasonable request.

## References

[1] Dc D. (2013). *Textbook of Obstetrics. Including Perinatology and Contraception*.

[2] Behrman R. E., Butler A. S., Institute of Medicine (2007). *Preterm Birth: Causes, Consequences, and Prevention*.

[3] WHO (2015). *Population division*.

[4] Chawanpaiboon S., Vogel J. P., Moller A.-B. (2019). Global, regional, and national estimates of levels of preterm birth in 2014: a systematic review and modelling analysis. *The Lancet Global Health*.

[5] Howson C. P., Kinney M. V., Lawn J. E. (2012). *Born Too Soon. The global action report on preterm birth*.

[6] USAID *Profile of Preterm and Low Birth Weight Prevention and Care*.

[7] Deribew A., Tessema G. A., Deribe K. (2016). Trends, causes, and risk factors of mortality among children under 5 in Ethiopia, 1990-2013: findings from the Global Burden of Disease Study 2013. *Population Health Metrics*.

[8] Mengesha H. G., Sahle B. W. (2017). Cause of neonatal deaths in Northern Ethiopia: a prospective cohort study. *BMC Public Health*.

[9] Al Riyami N., Al-Ruheili I., Al-Shezawi F., Al-Khabori M. (2013). Extreme preterm premature rupture of membranes: risk factors and feto maternal outcomes. *Oman Medical Journal*.

[10] Martin J. A., Hamilton B. E., Sutton P. D. (2013). Births: final data for 2007. *National Vital Statistics Reports*.

[11] Hodek J. M., von der Schulenburg J. M., Mittendorf T. (2011). Measuring economic consequences of preterm birth- methodological recommendations for the evaluation of personal burden on children and their caregivers. *Health Economics Review*.

[12] Tehranian N., Ranjbar M., Shobeiri F. (2016). The prevalence rate and risk factors for preterm delivery in Tehran, Iran. *Journal of Midwifery and Reproductive Health*.

[13] Ahumada-Barrios M. E., Alvarado G. F. (2016). Risk factors for premature birth in a hospital. *Revista latino-americana de enfermagem*.

[14] Mahajan A., Magon S. (2017). Study of risk factors for preterm births in a teaching hospital: a prospective study. *International Journal of Medical and Dental Sciences*.

[15] Teklay G., Teshale T., Tasew H., Mariye T., Berihu H., Zeru T. (2018). Risk factors of preterm birth among mothers who gave birth in public hospitals of central zone, Tigray, Ethiopia: unmatched case–control study 2017/2018. *BMC Research Notes*.

[16] Bekele I., Demeke T., Dugna K. (2017). Prevalence of Preterm Birth and its Associated Factors among Mothers Delivered in Jimma University Specialized Teaching and Referral Hospital, Jimma Zone, Oromia Regional State, South West Ethiopia. *Journal of Women's Health Care*.

[17] Rao C. R., de Ruiter L. E., Bhat P., Kamath V., Kamath A., Bhat V. (2014). Case-Control Study on Risk Factors for Preterm Deliveries in a Secondary Care Hospital, Southern India. *ISRN Obstetrics and Gynecology*.

[18] Passini R., Cecatti J. G., Lajos G. J. (2014). Brazilian multicentre study on preterm birth (EMIP): prevalence and factors associated with spontaneous preterm birth. *PLoS One*.

[19] Abaraya M., Seid S., Ibro S. (2018). Determinants of preterm birth at Jimma university medical center, Southwest Ethiopia. *Pediatric Health, Medicine and Therapeutics*.

[20] do Carmo Leal M., Esteves-Pereira A. P., Nakamura-Pereira M. (2016). Prevalence and risk factors related to preterm birth in Brazil. *Reproductive Health*.

[21] Gebreslasie K. (2016). Preterm Birth and Associated Factors among Mothers Who Gave Birth in Gondar Town Health Institutions. *Advances in Nursing*.

[22] Wagura P., Wasunna A., Laving A., Wamalwa D., Ng’ang’a P. (2018). Prevalence and factors associated with preterm birth at Kenyatta national hospital. *BMC Pregnancy and Childbirth*.

[23] Alijahan R., Hazrati S., Mirzarahimi M., Pourfarzi F., Hadi P. A. (2014). Prevalence and risk factors associated with preterm birth in Ardabil, Iran. *Iranian Journal of Reproductive Medicine*.

